# Nucleopolyhedrovirus Coocclusion Technology: A New Concept in the Development of Biological Insecticides

**DOI:** 10.3389/fmicb.2021.810026

**Published:** 2022-01-25

**Authors:** Trevor Williams, Miguel López-Ferber, Primitivo Caballero

**Affiliations:** ^1^Instituto de Ecología AC, Xalapa, Mexico; ^2^Hydrosciences Montpellier, Univ Montpellier, IMT Mines Alès, IRD, CNRS, Alès, France; ^3^Institute for Multidisciplinary Research in Applied Biology, Universidad Pública de Navarra, Pamplona, Spain; ^4^Bioinsectis SL, Noain, Spain

**Keywords:** baculovirus, genotypic variant, diversity, virus-virus interactions, complementation, insecticidal characteristics, host range

## Abstract

Nucleopolyhedroviruses (NPV, *Baculoviridae*) that infect lepidopteran pests have an established record as safe and effective biological insecticides. Here, we describe a new approach for the development of NPV-based insecticides. This technology takes advantage of the unique way in which these viruses are transmitted as collective infectious units, and the genotypic diversity present in natural virus populations. A ten-step procedure is described involving genotypic variant selection, mixing, coinfection and intraspecific coocclusion of variants within viral occlusion bodies. Using two examples, we demonstrate how this approach can be used to produce highly pathogenic virus preparations for pest control. As restricted host range limits the uptake of NPV-based insecticides, this technology has recently been adapted to produce custom-designed interspecific mixtures of viruses that can be applied to control complexes of lepidopteran pests on particular crops, as long as a shared host species is available for virus production. This approach to the development of NPV-based insecticides has the potential to be applied across a broad range of NPV-pest pathosystems.

## Introduction

Biological insecticides based on pathogenic microorganisms form part of the larger group of biorational insecticides that are selective, low-risk products and technologies that conserve natural enemy populations and present a reduced risk to non-target organisms and the environment (Ishaaya and Horowitz, 2009). The need for effective modern biorational products is driven by the increasing incidence of insecticide resistance in agricultural pests (Sparks and Nauen, 2015), increasing restrictions of the use of broad-spectrum compounds (Donley, 2019), and growing consumer demand for produce free of synthetic pesticide residues (Rana and Paul, 2017). Due to their natural origin, biosafety characteristics and compatibility with organic agriculture, biological insecticides can provide unique tools for pest control that are well accepted by growers and consumers.

Lepidopteran nucleopolyhedroviruses (NPVs) (Family *Baculoviridae*, genus *Alphabaculovirus*) are virulent pathogens that lethally infect the larval stages of these insects, including many species of pests. Natural isolates of these viruses have been used as the active ingredients of biological insecticides in developed and developing countries worldwide (Moscardi et al., 2011; Haase et al., 2015; Sun, 2015). These products have highly selective insecticidal characteristics, an outstanding environmental profile and a clear record of safe use in pest control established over many decades, although their high host specificity and costs of mass production are considered important limitations to their wide-scale use as insecticides (Lacey et al., 2015). Previous attempts at improving the insecticidal characteristics of these pathogens have mainly focused on increasing their speed of kill in order to reduce the crop feeding damage inflicted by the pest (Moscardi et al., 2011). This has been achieved by insertion of neurotoxin genes (Inceoglu et al., 2001; Kamita et al., 2005) or by deletion of virus genes that extend insect lifespan (O’Reilly and Miller, 1991). However, the mass-production of genetically modified viruses faces important limitations and many countries prohibit the use of genetically modified organisms in agriculture.

In the present review, we describe a new approach to the development of the active ingredient of NPV-based insecticides that we have successfully employed over the past decade. The technology involves taking advantage of the unique way in which these viruses are transmitted and the genotypic diversity present in natural populations of the viruses. Most recently, we have adapted the technology to produce custom-designed mixtures of viruses that can be applied to control complexes of lepidopteran pests on particular crops. This technology has the potential to be applied across a broad range of NPV-pest pathosystems.

## Basic Biology and Replication Cycle of Nucleopolyhedroviruses

**FIGURE 1:**
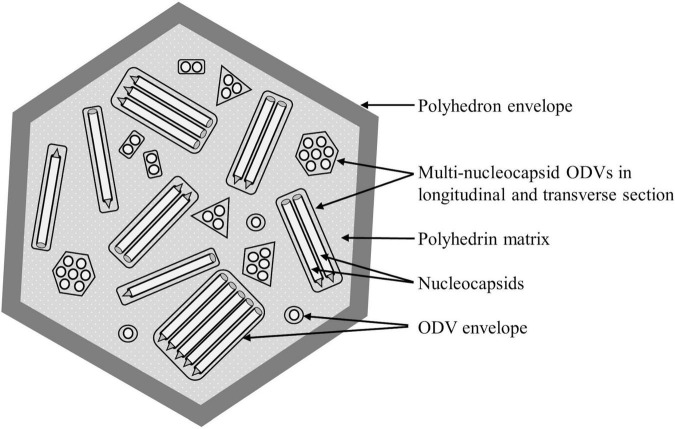
Schematic diagram of the structure of a multiple nucleopolyhedrovirus occlusion body (OB). Rod-like viral nucleocapsids each containing a single virus genome are enveloped singly or in groups into occlusion derived virions (shown in longitudinal and transverse section) that are occluded within a matrix of crystalline polyhedrin protein and wrapped by a smooth polyhedron envelope.

Lepidopteran NPVs are large occluded viruses with a circular double-stranded DNA genome of 80–180 kbp (Harrison et al., 2018). Each genome is packaged within a rod-shaped nucleocapsid (Figure 1). The nucleocapsids are enveloped singly or in groups to form occlusion derived virions (ODVs). The ODVs are in turn occluded within a crystalline matrix of polyhedrin protein, covered with a smooth external polyhedron envelope protein (PEP) to form a polyhedral occlusion body (OB), typically 1–2 μm in diameter, that protects the ODVs in the environment (Sajjan and Hinchigeri, 2016). There are two morphotypes of NPVs; the more common multiple nucleocapsid NPVs in which ODVs comprise groups of one to many nucleocapsids (shown in Figure 1), and the less common single nucleocapsid NPVs, in which each nucleocapsid is enveloped singly. In both cases, however, numerous ODVs are occluded within each OB.

Transmission occurs when a larva of a susceptible species consumes plant foliage contaminated with OBs. In the insect midgut, the alkaline pH dissolves the OBs releasing the ODVs that cross the peritrophic matrix and infect midgut epithelial cells (Erlandson et al., 2019). The ODVs are structurally complex and comprise many proteins, including the *per os* infection factors (PIFs) that form a protein complex that is essential to gain access to the host cell during the primary infection process (Wang et al., 2019). Initial replication occurs in the cell nucleus and results in the production of budded viruses, each containing a single nucleocapsid. These virions bud out of the cell and disperse *via* the hemolymph and the insect tracheal system to infect the cells of other tissues (Passarelli, 2011). Later in infection, ODVs are formed and are occluded within the cell nucleus to form OBs. Shortly before death, infected larvae often climb to the upper parts of the host plant and die characteristically suspended from the prolegs. Viral enzymes degrade the larval integument releasing millions of progeny OBs that contaminate foliage for the following cycle of transmission (Williams, 2018).

## Nucleopolyhedrovirus Natural Isolates Are Mixtures of Variants

Variation in NPVs is observed both among natural isolates from different infected insects and within each virus killed insect. Using *in vivo* and *in vitro* cloning techniques, over twenty genotypic variants have been purified from individual NPV-infected insects (Cory et al., 2005; Baillie and Bouwer, 2012). *In vitro* cloning usually involves collecting budded virions in the hemolymph of infected larvae, followed by dilution and plaque purification or end-point dilution using established cell culture techniques (O’Reilly et al., 1993). *In vivo* cloning involves serial inoculation of larvae with very low doses of OBs, or injection of larvae with low concentrations of budded virions from the hemolymph of an infected insect. These techniques can give quite different results in terms of the diversity and characteristics of the purified genotypes, due to the divergent conditions required for replication and transmission in insects compared to cell culture systems (Erlandson, 2009).

Characterization of genetic diversity in baculoviruses has often involved the use of restriction endonuclease enzymes, whereas recent studies increasingly employ next-generation genome sequencing. The diversity present in baculovirus genomes consists of numerous single nucleotide polymorphisms (SNPs) spread across the genome and insertions, deletions, inversions and duplications that are frequently located around hotspots such as *hoar*, *hrs* and *bro* genes (Erlandson, 2009; Brito et al., 2016; Masson et al., 2021). Significant variation has also been observed in the viral DNA polymerase (*dpol*) gene (Kitchin and Bouwer, 2018), and in genes encoding ODV envelope proteins including PIFs and ODV-E66 that are critical for primary infection of midgut cells (Simón et al., 2011; Craveiro et al., 2013; Thézé et al., 2014), and also in non-essential auxiliary genes such as *egt, chitinase* and *enhancin* that improve OB production and transmission (D’Amico et al., 2013; Harrison, 2013; Martemyanov et al., 2015; Niz et al., 2020; Masson et al., 2021).

In other cases, structural variants with large deletions, sometimes representing over 10% of the genome, are present in up to one third of all variants in some NPV populations (Simón et al., 2004; Redman et al., 2010; Barrera et al., 2011; Chateigner et al., 2015; Loiseau et al., 2020). For example, structural variation is present in 40% of Autographa californica multiple nucleopolyhedrovirus (AcMNPV) genomes, but structural variants are almost always eliminated in serial passage due to their negative effects on virus fitness (Loiseau et al., 2020). Variants with large deletions can only persist in the presence of complete genotypes that encode the missing gene products in cells coinfected by complete and deletion genotypes. This process of complementation among variants is possible because during systemic infection, each cell is infected by approximately four budded virions, each of which may be a deletion variant or a complete genotype (Bull et al., 2001). Coinfection of cells by multiple genotypes also provides opportunities to generate diversity through recombination (Kondo and Maeda, 1991; Kamita et al., 2003).

The DNA polymerases of NPVs have proofreading activity, which implies a high degree of fidelity during DNA replication (Mikhailov et al., 1986; Hang and Guarino, 1999; McDougal and Guarino, 1999). Indeed, a recent estimate of the rate of mutation during replication, in the absence of selection bias, indicated that AcMNPV has a mutation rate of approximately 1 × 10^–7^ substitutions per nucleotide per round of copying, typical of the very low mutation rates observed in DNA viruses (Boezen et al., 2021). In spite of this, single nucleotide polymorphisms are present at high frequency (∼10^–3^) in NPV populations (Chateigner et al., 2015; Masson et al., 2021), and some variants are capable of generating remarkable levels of variation spontaneously during replication, a process that is particularly apparent when host larvae are inoculated with low doses of OBs (Baillie and Bouwer, 2013; Aguirre et al., 2021).

## Why So Much Variation?

The fact that genetic diversity is maintained and transmitted among hosts indicates that such variation is selectively advantageous to the virus. Specifically, genetic variation in virus populations is favored by heterogeneity in host susceptibility to infection (van der Werf et al., 2011; Fleming-Davies et al., 2015; Hudson et al., 2016), differential transmission of variants on food plants (Hodgson et al., 2002; Raymond et al., 2002), and host species-mediated selection in the case of NPVs that infect multiple host species (Hitchman et al., 2007; Zwart et al., 2009).

In addition, genotypic heterogeneity in virus populations may provide a source of preadaptation that allows the pathogen to exploit a range of host genotypes or overcome variation in the characteristics of different host foodplant species (Hodgson et al., 2002; Raymond et al., 2002) and different plant structures (Fuxa, 2008). Variant diversity also provides opportunities for risk-spreading in response to environmental stochasticity, such as the presence of genotypes with divergent tendencies for vertical or horizontal transmission that may be differentially favored as host densities fluctuate (Cabodevilla et al., 2011).

Individual genotypic variants usually vary markedly in OB dose-mortality metrics, speed of kill and OB production characteristics (Cory et al., 2005; Erlandson, 2009). These traits clearly contribute to virus transmission and the efficiency with which the virus converts host resources into virus progeny. However, they are constrained by correlations and tradeoffs, such as that of speed of kill and OB production (Hunter-Fujita et al., 1998; Simón et al., 2012). Fast-killing variants kill the host shortly following infection so that each insect represents an almost fixed resource for OB production, whereas larvae infected with slow-killing variants continue to feed and grow during the infection incubation period, thereby providing additional resources for virus production. Rapid speed of kill also proved to be costly to OB persistence and transmission in the *Lymantria dispar*—LdMNPV pathosystem, indicating *trans-*generational effects on the fitness of fast-killing variants (Fleming-Davies and Dwyer, 2015).

## How Is Variant Diversity Transmitted?

The unusual structure of OBs and ODVs has unique implications for the transmission of NPV genomes. In the multiple-nucleocapsid type ODVs, nucleocapsids carrying potentially different genotypes (variants) are enveloped together in groups of 1–29 nucleocapsids per ODV (Adams and McClintock, 1991). Consequently, fusion between the midgut cell membrane and the ODV membrane often results in the delivery of multiple genomes, and potentially multiple variants into the host cell. An additional layer of complexity comes from the occlusion of ODVs within each OB. The OB is the unit of dosage and each OB occludes dozens of ODVs. As an insect consumes a certain number of OBs when feeding on a host plant, even at the minimum dose of a single OB, the host insect receives an inoculum comprising many nucleocapsids, which provides a mechanism for the simultaneous transmission and coinfection of the host by multiple genotypic variants (Clavijo et al., 2010). Dosing of larvae with individual OBs is now possible following the development of highly accurate laser capture microdissection techniques that show considerable promise for future studies on the virulence and genetic diversity of single OBs (Munsamy and Bouwer, 2020).

Cooperative social interactions among viruses can have clear benefits for their transmission, replication, and ability to overcome host immune defenses (Díaz-Muñoz et al., 2017; Sanjuán, 2021). For example, viruses that are transmitted in groups represent “collective infectious units” (Sanjuán, 2017). For NPVs, both OBs and multi-nucleocapsid ODVs clearly represent collective infectious units that have a series of potential advantages for the virus:

(i) The structure of the OB itself allows the virus to persist and disperse in the environment, in soil or plant surfaces, for extended periods until consumed by a susceptible larva. This allows virus survival during periods of low host density, including overwintering (Fuxa et al., 2001; Fuller et al., 2012).
(ii) Multi-nucleocapsid ODVs deliver various nucleocapsids to the host cell, a fraction of which may uncoat in the cell nucleus and initiate replication, whereas others can travel to the basal membrane, acquire the GP64 protein (group I NPVs) that is produced early in infection. These nucleocapsids can then leave the cell as budded virus to establish a rapid systemic infection that does not depend on the destiny of the primary infection of the midgut cell (Granados and Lawler, 1981; Washburn et al., 1999). In effect, this is a bet-hedging strategy by which the virus reduces the risk of stochastic events that could eliminate the founder population at the initial stages of infection (Sanjuán and Thoulouze, 2019).
(iii) Simultaneous infection by multiple virus genomes could provide a mechanism of cooperative positive feedback between virus genome templates and the virally encoded products that promote replication (Andreu-Moreno et al., 2020), although this has not been investigated in NPVs.
(iv) A group of virus genomes may be better able to overcome the innate immune response of the host cell (Sanjuán and Thoulouze, 2019). As NPVs carry an arsenal of anti-apoptotic and global-protein-shutdown-blocking genes (Nagamine, 2021), the presence of multiple copies of these genes at the earliest stages of infection could improve the likelihood of a productive infection (Ikeda et al., 2013).
(v) The composition of the genotypes present in an OB or an ODV determines the diversity of variants that infect a new host. As such, genotypically diverse OBs provide a mechanism for alleviating the genetic bottleneck that inevitably occurs when larvae consume low doses of inoculum (Sanjuán, 2017). For NPVs, this bottleneck has been estimated at 1.3–6.3 virions/insect depending on inoculum dose and insect stage (Zwart and Elena, 2015). Consumption of a single OB by a larva of *Spodoptera frugiperda* was demonstrated to result in the transmission of between one and five genotypic variants, including rare variants that comprised less than 1% of the virus inoculum (Clavijo et al., 2010). Coenveloping of genotypic variants within ODVs therefore provides a mechanism for the survival of infrequent variants.

The transmission of NPVs in collective units has a clear genetic basis and is therefore a trait that is subject to selection. Polyhedrin, P10 and the polyhedron envelope protein (PEP) are the main structural components of the viral OB (Rohrmann, 2019). These proteins tend to be highly conserved among NPVs. Of the genes involved in ODV composition, *ac23* (F-protein), *ac78* and *sf32* have been demonstrated to influence the number of nucleocapsids (genomes) enveloped in each ODV (Yu et al., 2009; Beperet et al., 2013; Li et al., 2014). In contrast, *ac78*, *ac132* and *sf29* influence the number or type of ODVs occluded within OBs (Simón et al., 2008; Li et al., 2014; Yang et al., 2014), whereas *fp25k* (*ac61*) affects the occlusion of ODVs in cell culture conditions (Cheng et al., 2012). Of these genes, only *sf29*, a putative collagenase, has been identified as undergoing diversifying selection (Masson et al., 2021).

## Intraspecific Coocclusion as a Method of Producing Improved Insecticides

Here, we propose the coocclusion of laboratory-controlled selected combinations of genotypic variants as a novel strategy for the development of NPV preparations with improved insecticidal activity. This approach originated from the observation that a natural Nicaraguan isolate of Spodoptera frugiperda multiple NPV (SfMNPV) comprised nine different genotypic variants. Of these, three defective variants were not infectious when administered perorally to larvae because they lack the genes for certain PIF factors that are essential for infection in the midgut of the larval host. These defective variants were able to take advantage of the cellular pool of virally encoded proteins produced by the other variants present within the cell, including the PIF factors (López-Ferber et al., 2003; Simón et al., 2013). OBs comprising the only complete genotypic variant (variant B) were threefold less pathogenic than the wild-type isolate OBs. However, when mixed with one of the defective variants (variant C) in a ratio of 3:1 (B:C) and cooccluded by coinfection of larvae, the resulting OBs comprised a mixture of both variants in a ∼3:1 ratio and OB pathogenicity was restored to that of the wild-type isolate.

These findings indicated that interactions among cooccluded variants (that by definition must have replicated in the same host cell) could alter the phenotype of progeny OBs. Additional studies with this pathosystem revealed that the number and identity of variants in cooccluded mixtures and their ratio resulted in mixtures with altered OB pathogenicity, speed-of-kill and OB production characteristics (Simón et al., 2005, 2008).

**FIGURE 2:**
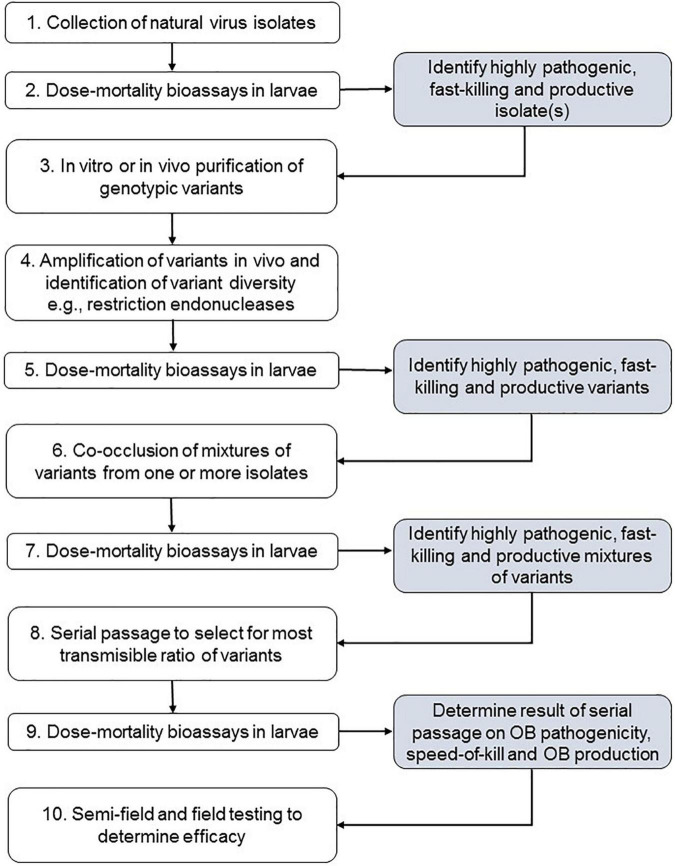
Methodological steps (white boxes) and objectives (gray boxes) required for application of variant coocclusion technology to the production of nucleopolyhedrovirus preparations with improved insecticidal characteristics.

These findings led us to design the following ten-step pathway for the development of the active material for NPV-based insecticides (Figure 2).

**FIGURE 3:**
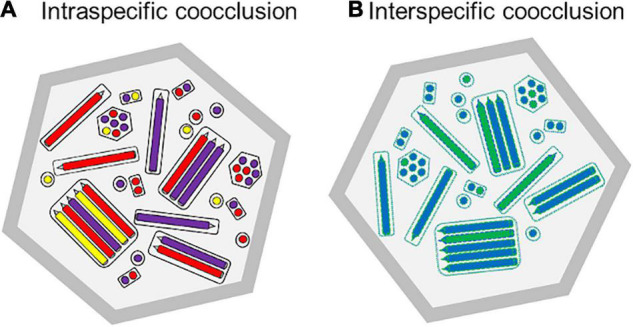
Theoretical schematic diagram of the cross-section of occlusion body preparations that comprise: **(A)** Intraspecific mixture of three genotypic variants of a multiple nucleopolyhedrovirus, represented as purple, red and yellow nucleocapsids in longitudinal and transverse section, and **(B)** Interspecific mixture of two different nucleopolyhedroviruses, shown as blue and green nucleocapsids. As these viruses replicated in the same cell, the nucleocapsids, tegument, ODV envelope associated proteins and PIF proteins would comprise mixtures of proteins produced by both viruses (shown as green and blue dashed lines).

(i) Briefly, this involves access to a collection of natural NPV isolates from the insect pest of interest (step 1).
(ii) Dose or concentration-mortality bioassays are then performed in the target host to identify the most pathogenic, fast-killing and productive natural isolates available (step 2).
(iii) The selected isolates are then subjected to purification in a suitable cell line (plaque purification) or by *in vivo* cloning using very low concentrations of BV inoculum injected into larvae over several rounds, although this is a laborious procedure compared to plaque purification (step 3). Importantly, cell culture is likely to select for variants that are particularly amenable to replication in an *in vitro* system, whereas *in vivo* cloning of BVs replicates natural conditions, except for the critical gut infection process.
(iv) Sampling of a large number of isolated plaques in combination with a genotype characterization procedure, such as the use of restriction endonuclease profiles, allows the classification of genotypic variants according to their prevalence in the natural isolate population. It is usually necessary to amplify plaque picks by injection into larvae to produce sufficient genomic DNA for restriction enzyme analysis (step 4).
(v) Each identified variant (typically 1–24 variants per isolate) is then subjected to insect bioassay to determine OB pathogenicity, speed of kill and OB production characteristics (step 5).
(vi) Variants with particularly interesting insecticidal traits can then be selected for coocclusion studies. At this stage it is useful to acquire genome sequence information for the variable regions so that qPCR primers can be designed for subsequent quantification of laboratory-controlled mixtures of the variants. For coocclusion, pure variant OBs can be mixed and used for peroral inoculation of larvae in different ratios for coocclusion of the selected combinations of variants (step 6). The use of high inoculum concentrations or doses ensures the infection of larvae by variants that differ in OB pathogenicity. Alternatively, if variants have no or very low peroral infectivity, the injection of known mixtures of variant BVs into host larvae allows variant replication in coinfected cells and, as a result, the production of cooccluded mixtures of variants.
(vii) Once cooccluded mixtures of variants have been produced (Figure 3A), their insecticidal characteristics are compared by insect bioassay (step 7).
(viii) Following this, the most promising combinations are subjected to serial passage in larvae at an intermediate inoculum concentration, such as a 50% lethal concentration of OBs (step 8).
(ix) Serial passage involves inoculating larvae and collecting the OBs from those that die from lethal infection. These OBs are then used as the inoculum for the following round of inoculation. In essence, this is a process of artificial selection for variants in the most transmissible proportions. Between four and six steps of serial passage can result in marked changes in the proportions of the variants present in the cooccluded mixture. These changes can be readily quantified by qPCR. Following this procedure, the resulting OBs are subjected to insect bioassay to determine the improvement, if any, in pathogenicity, speed-of-kill and OB production characteristics of the passaged OBs (step 9).
(x) Finally, the selected combination of variants can be subjected to greenhouse and field testing to determine the efficacy of the preparation compared to reference biorational products, such as insect growth regulators, spinosyns or insecticides based on *Bacillus thuringiensis* (step 10).

Interactions among genotypes can involve both *cis* and *trans* acting factors. An example of a *trans* effect is that of viral enhancins, located in the OBs, which are released in the midgut as OBs dissolve (Slavicek, 2012). These enzymes degrade the peritrophic matrix allowing ODVs to pass through and infect midgut cells, even if these ODVs originate from different OBs. The enhancin factor is separate and independent of the virions. Conversely, for *cis*-acting factors a physical association with the virion is required. Such interactions occur among different variants during coinfection of individual cells and the sharing of the products of transcription as a pool of public goods. This results in ODVs with a pseudotype that reflects the genetic composition of the cell in which they were produced. Hence, by coinfection, virions carrying the genomes of defective variants are able to acquire the infectious pseudotype of the viable variants alongside which they replicated. Consequently, we expect *cis*-type interactions to occur in single nucleocapsid and multiple nucleocapsid NPVs and also in granuloviruses (genus *Betabaculovirus*) (Harrison et al., 2018). As a result, the coocclusion of variants *per se* is not strictly required for transmission of rare or unusual variants, such as defective variants, as long as they previously shared a host cell with one or more viable genotypes that could provide the necessary proteins for virion pseudotyping.

In reality, for multiple type NPVs, variants that replicate together will most likely be enveloped together in ODVs and cooccluded in mixed variant OBs. In contrast, for single type NPVs in which nucleocapsids are enveloped individually, the OB, containing numerous such ODVs, defines the collective infectious unit of transmission. The situation is different for granuloviruses, as each granulovirus OB usually occludes only a single ODV comprising a single nucleocapsid (Tanada and Hess, 1991), although rare exceptions have been reported (Falcon and Hess, 1985; Vargas-Osuna et al., 1994; Sciocco-Cap et al., 2001). As such, granuloviruses are not usually transmitted as collective infectious units, and the probability of coinfection of an insect will depend entirely on the genetic diversity distributed across inoculum OBs and the number of OBs consumed.

The application of the intraspecific coocclusion procedure can best be exemplified by two examples.

### Example 1: Coocclusion of ChchNPV Variants

The Chysodeixis chalcites nucleopolyhedrovirus (ChchNPV) is a single type NPV in which ODVs comprise a single nucleocapsid with a single genome. Coocclusion of selected variants of ChchNPV resulted in a faster killing and more pathogenic preparation than a wild-type isolate from Tenerife, Spain (Bernal et al., 2013b). An initial study involving 97 natural isolates of ChchNPV from banana plantations in the Canary Islands revealed that isolates could be classified into one of five groups based on their restriction profiles (Bernal et al., 2013a). The most prevalent profile, named ChchNPV-TF1, represented 78% of the isolates examined. Notably, ChchNPV-TF1 was 15- to 20-fold more pathogenic in terms of OB potency than any other Canary Island isolate or reference isolates from Netherlands or southern Spain, and was slightly faster killing than the other isolates (Bernal et al., 2013a).

When ChchNPV-TF1 was subjected to plaque purification in permissive High-Five cells, the natural isolate was found to comprise a mixture of eight genotypic variants that could be distinguished by their BglII restriction profiles (Bernal et al., 2013b). Genome sequencing revealed that most of the sequence variation was localized in the *hoar* and *bro-d* genes (Bernal et al., 2013c). Quantitative PCR analysis indicated that the prevalence of the most common variants in ChchNPV-TF1, named A, B and C, accounted for 76% of the isolated clones, whereas the five other variants comprised between 1 and 11% of the variants present in the natural isolate (Table 1). The OB potency of each of the individual variants was low (<0.2 in all cases) compared to the natural isolate. However, coocclusion of variants A, B and C in the ratio in which they were present in the wild type isolate (47:34:19 for A, B and C, respectively) resulted in a twofold increase in OB potency and a ∼30% faster speed of kill compared to the wild isolate (Table 1). The OB production values in *C. chalcites* second instars were significantly reduced in the cooccluded mixture compared to the natural isolate, presumably due to the rapid speed of kill of this mixture. A serial passage experiment revealed that the cooccluded mixture was stable over five steps of *in vivo* passage and did not change significantly in OB potency characteristics (Bernal et al., 2013b).

**TABLE 1:** Characteristics of nucleopolyhedrovirus natural isolates used for variant selection and coocclusion.

Virus	No. isolates analyzed	Relative potency of most insecticidal isolate OBs	No. genotypic variants identified	Relative potency of OBs of individual variants	No. of variants cooccluded	Relative potency of cooccluded mixture of variants	Speed of kill of cooccluded mixture of variants	OB production of cooccluded mixture of variants	Effect of serial passage on cooccluded mixture of variants
ChchNPV	97	15.5	8	0.09–0.18	3	2.1	∼30% faster	∼40% reduction	Stable, no change in OB potency
HearNPV	20 + 17	2.8	8	1.0–2.8	2	6.3	No change	0–28% reduction	Shift in favor of SP1B variant; 1.7-fold increase in OB potency

*Coocclusion resulted in changes in occlusion body (OB) potency, speed of kill and OB production, further modified by serial passage in vivo.*

### Example 2: Coocclusion of HearNPV Variants

HearNPV is a single nucleocapsid type NPV. A survey of 20 natural isolates from maize and tomato crops in Spain and Portugal revealed the presence of eight groups that differed in their restriction profiles (Figueiredo et al., 2009; Arrizubieta et al., 2014). The most pathogenic strain (SP1) was 2.8-fold more potent than a reference isolate from China (HearNPV-G4) and was also the fastest killing isolate (Arrizubieta et al., 2015). Plaque purification of SP1 resulted in the isolation of two genotypic variants (SP1A, SP1B). The SP1B variant was 2.8-fold more pathogenic than the natural isolate SP1. End-point dilution of 17 natural isolates from cotton resulted in the isolation of six genotypic variants, named LB1- LB6, that had a relative potency similar to that of the SP1 isolate (Table 1).

Eight cooccluded preparations were produced comprising eight different mixtures of variants in various proportions. Bioassays revealed that a binary mixture of SP1B + LB6 (1:1 ratio) had an OB potency 6.3-fold higher than that of the SP1 wild isolate, although with a slightly (6%) slower speed of kill (Arrizubieta et al., 2015). Serial passage of the binary mixture (SP1B + LB6) in larvae led to a marked shift in favor of the SP1B variant that reached a prevalence of 85% in the mixture after five passage steps. This change was associated with an additional 1.7-fold increase in OB potency and a ∼12% faster speed of kill of the passaged preparation (Table 1). However, OB production was significantly reduced following serial passage. Quantitative PCR analysis of *H. armigera* larvae inoculated with a single OB confirmed that both variants were cotransmitted in all larvae tested (Arrizubieta et al., 2015). Subsequent field testing of the binary mixture on tomato crops indicated that this preparation was as effective in crop protection as spinosad or an insecticide based on *Bacillus thuringiensis* (Arrizubieta et al., 2016). Potentiation among variants of the single nucleocapsid HearNPV provides an example of how coinfection of cells and use of the shared pool of proteins can generate the observed phenotypic changes in variant mixtures.

### Interspecific Coocclusion for the Production of Insecticides With Broader Host Range

As mentioned previously, the narrow host range of NPVs limits their use in crops attacked simultaneously by several species of pests (Lacey et al., 2015). Following advances in the coocclusion of virus variants, recently it was found possible to coocclude mixtures of different species of NPVs (Beperet et al., 2021). Obviously, for this, the viruses selected had to be able to replicate in a shared host species. Four cooccluded preparations were produced involving (i) SfMNPV + AcMNPV (99.95:0.05% composition), (ii) AcMNPV + MbMNPV (35:65%), (iii) SfMNPV + MbMNPV (53:47%), all of which replicated in *S. frugiperda* larvae, and (iv) SeMNPV + SfMNPV (95:5%) which replicated in *S. exigua* larvae. As host susceptibility to each virus and the replication efficiency of each virus differed, the composition of the cooccluded mixture was adjusted by altering the doses of OBs of each virus used to inoculate larvae, or by inoculating larvae with the heterologous virus followed 24 h later by the homologous virus (Beperet et al., 2021). In the case of the SeMNPV + SfMNPV mixture, a defective variant of SfMNPV was used that lacked two per os infection factors (PIF-1, PIF-2) in the ODV envelope that are essential for peroral infection of midgut cells. In this case, a mixture of ODVs was injected into larvae to initiate infection. By coinfecting cells together with SeMNPV, the defective variant of SfMNPV was able to persist over six cycles of peroral passage in *S. exigua* larvae, albeit at decreasing prevalence, despite lacking the *pif-1* and *pif-2* genes. The defective variant was cotransmitted with SeMNPV due to the presence of PIF factors produced by SeMNPV in cells infected by both viruses. A subsequent study demonstrated that the asynchronous inoculation of larvae at intervals of 12–72 h resulted in marked changes in the composition of the cooccluded mixture of viruses (Beperet-Arive, 2014). This was due to a block to superinfection by a second virus established 12–16 h after infection by the first virus, a phenomenon known as superinfection exclusion that was concurrent with the reorganization of the actin cytoskeleton in virus-infected cells (Beperet et al., 2014).

**FIGURE 4:**
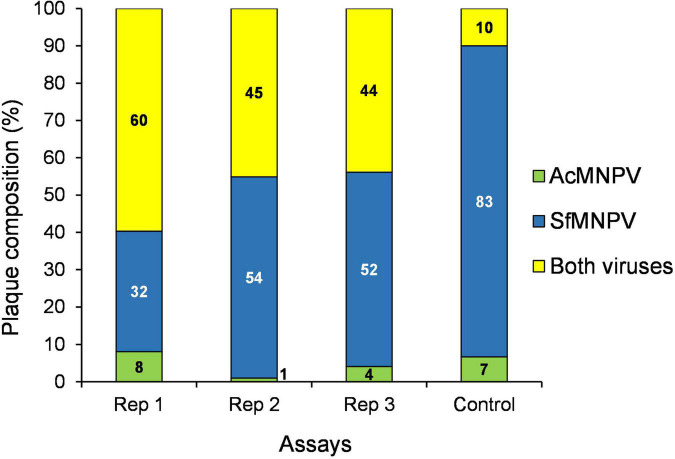
Composition of occlusion derived virions (ODVs) in a mixed virus cooccluded preparation of SfMNPV + AcMNPV (99.95:0.05%). Interspecific coocclusion was achieved by inoculation of *Spodoptera frugiperda* larvae. Prevalence of single virus and mixed-virus plaques produced following inoculation of ODVs in permissive Sf9 cells. Plaques were classified as single or mixed-virus by qPCR analysis. Three independent virus preparations were analyzed (Rep 1–3). The control preparation consisted of a mixture of pure SfMNPV ODVs and pure AcMNPV ODVs that were mixed prior to plaque purification to control for adhesion among ODVs and contamination events during the assays. Mixed-virus plaques did not exceed 10% in the control. Values within columns indicate percentages of plaques in each category [Figure modified from Beperet et al. (2021)].

Plaque purification and end-point dilution of ODVs released from mixed-virus OBs revealed that the viruses had been coenveloped to form mixed-virus ODVs prior to coocclusion (Figure 4). For example, when the AcMNPV + SfMNPV cooccluded preparation was subjected to plaque purification in Sf9 cells that are permissive to both viruses, plaque analysis by qPCR demonstrated that between 44 and 60% of plaques comprised both viruses (Figure 4). This suggests that approximately half of the ODVs used as inoculum will have comprised interspecific mixtures of genomes. This was a higher proportion than expected, given that qPCR indicated that just 0.05% of the genomes present in ODVs were those of AcMNPV. This likely reflects the high affinity to cell culture of AcMNPV, so that SfMNPV genomes that had been coenveloped in ODVs with AcMNPV were more likely to infect cells *in vitro* due to the presence of AcMNPV proteins in the ODV envelope (Figure 4). The example of the SeMNPV + SfMNPV preparation also demonstrates that ODVs produced in coinfected cells comprise interspecific mixtures of proteins, such as PIFs, from both of the viruses that replicated in each infected cell (Figure 3B). The mixed-virus coocclusion technology was patented (Caballero-Murillo et al., 2018).

An additional laboratory demonstration of the value of the technology came from a study in which the single nucleocapsid HearNPV was cooccluded with the multinucleocapsid nucleopolyhedrovirus, HearMNPV. The cooccluded mixture was able to infect, replicate and persist during insect serial passage in two host species that are not susceptible to HearNPV alone, again as a result of the sharing of pooled proteins in coinfected cells (Arrizubieta Celaya, 2015). Such findings suggest that this technology has clear potential for producing non-recombinant baculovirus preparations with a widened host range that could be applied for the control a broader range of insect pests than possible using NPV-insecticides based on a single virus.

## Future Perspectives

The advances described in the present review open the way to a diversity of future studies, both as a means to improving the efficacy of NPV-based insecticides and as a research tool to better understand novel aspects of host-virus and virus-virus interactions in NPV-based pathosystems. Potential improvements to NPV-based insecticides include the possible potentiation of the insecticidal properties of mixtures of viruses, as observed when mixtures of variants are cooccluded. The use of virus mixtures and the findings that mixtures of HearNPV and HearMNPV resulted in altered host range suggest that this technology could provide a means to control complexes of lepidopteran pests in certain crops. It may also allow a broader range of host species to be used for the production of mixed-virus preparations, although the impact of the use of alternative hosts on the insecticidal characteristics of the resulting preparation would require careful evaluation.

The industrial production of mixed-variant OBs could be achieved following established procedures (Grzywacz et al., 2014), although careful quality control would be necessary to ensure that variant composition remains stable over time and across different batches of insects. In contrast, mass-production of mixed-virus OBs represents a challenge given that a susceptible host for both viruses needs to be identified, and serial passage of inoculum results in the rapid decline of the slower replicating, or less virulent virus in the mixture. To address this, large-scale inoculation of larvae would have to involve the periodic preparation of a large stock of inoculum and avoid serial passage of any mixed-virus inocula.

The need for full testing of the efficacy of virus mixtures to control complexes of pests in the field is also a priority for establishing which pests and pest complexes are most amenable to control by mixed-virus technology. This approach has begun to be studied by others, for example for the control of lepidopteran pests in soybean (Sanches et al., 2019, 2021). Some manufacturers currently provide mixtures of OBs of different variants to overcome resistance in the pest population (Gueli-Alletti et al., 2017). Other manufacturers mix OBs of different viruses, presumably produced in different host species, to target complexes of pests (Anonymous, 2019). We suggest that the use of cooccluded preparations might have two advantages. First, production costs might be reduced as only one susceptible host insect is required for production of each virus mixture. Second, as described in this review, virion pseudotyping during coinfection will result in ODVs with altered infective capacities that may favor their use as biological insecticides, although this remains to be verified in field testing.

From a research perspective, proteomic studies on the composition of pseudotyped viruses produced in coinfected hosts could contribute to defining the influence of ODV or BV composition on both virion infectivity and the virion-associated factors that determine host range.

Finally, this review explored the interactions between viruses infecting the same cell, i.e., *cis* interactions. Nonetheless, other possible interactions could occur between viruses infecting the same host, but different cells, in a *trans* interaction mode. For NPVs, the success of primary infections appears to rely on the former, but granuloviruses probably use the latter, possibly mediated through cells infected by helper genotypes, which release a diffusible factor that circulates within the infected larva (Hinsberger et al., 2021). Future studies will doubtless provide additional evidence of the importance of intra- and interspecific social interactions among viruses (Sanjuán, 2021), and aid the growing recognition of the value of sociovirological approaches to understanding these pathogens.

## Author Contributions

TW wrote the manuscript. PC and ML-F edited and improved the manuscript. All authors conceptualized the review, contributed to the article, and approved the submitted version.

## Conflict of Interest

Bioinsectis SL is a spinoff company of the Universidad Pública de Navarra that is currently evaluating the commercial applications of the coocclusion technology described in this review. The authors declare that the research was conducted in the absence of any commercial or financial relationships that could be construed as a potential conflict of interest.

## Publisher’s Note

All claims expressed in this article are solely those of the authors and do not necessarily represent those of their affiliated organizations, or those of the publisher, the editors and the reviewers. Any product that may be evaluated in this article, or claim that may be made by its manufacturer, is not guaranteed or endorsed by the publisher.
